# Supplementary material for: Multidrug-resistant bacterial profiles of inanimate objects at Zewditu Memorial Hospital, Addis Ababa, Ethiopia
Source: PLoS One. 2025 Oct 7;20(10):e0333361. doi: 10.1371/journal.pone.0333361 (PMC12503313; doi:10.1371/journal.pone.0333361)
Supplement: Attachment — Submitted filename: PONE-D-25-12506 reviewer report.pdf [file pone.0333361.s002.pdf]

Manuscript Number: PONE-D-25-12506

Title: Multidrug-resistant bacterial profiles of inanimate objects at Zewditu Memorial Hospital, Addis Ababa, Ethiopia.

Overview and general recommendation:

This subject is **very important** because in developing countries, there is a lack of information on the extent and types of contamination, as well as the microbiological profiles of commonly used medical instruments and inanimate surfaces in hospitals. Therefore, ongoing research in this area is necessary to address and mitigate the problem.

I found the manuscript well written, well described and the references were new. I listed **minor points** below for editing.

Comments to Authors:

Minor points

1- **In this manuscript:** you wrote **(12 times Our)!!!** The rule of manuscript writing is to avoid using **(Our).** So you should delete **(Our),** use the **formal scientific words (This review or The current review or The present review).**

2- **In 364 line;** you wrote **(We)!!!** The rule of manuscript writing is to avoid using **(We).** So you should delete **(We),** use the **formal scientific words (This review or The current review or The present review).**

3- **In discussion: 366line;** I very recommend you to add new references here related with [Hospital-acquired infections and carbapenemase-producing isolates with gene transfer or mutations] to be stronger scientifically. Kindly add the two mentioned new references

Hasan SA, Raoof WM, Ahmed KK. Antibacterial activity of deer musk and Ziziphus spina-christi against carbapebem resis-tant gram negative bacteria isolated from patients with burns and wounds. Regulatory Mechanisms in Biosystems. 2024 Apr 17;15(2):267-78. [**DOI:** https://doi.org/10.15421/022439]

Hasan SA, Raoof WM, Ahmed KK. FIRST REPORT OF CO-HARBORING BLEOMYCIN RESISTANCE GENE (bleMBL) AND CARBAPENEMASE RESISTANCE GENE (blaNDM-1) KLEBSIELLA PNEUMONIAE IN IRAQ WITH COMPARISON STUDY AMONG THE SENSITIVITY TEST, THE BD PHOENIX CPO DETECT TEST, AND THE RAPIDEC® CARBA NP TEST. Siberian Journal of Life Sciences and Agriculture. 2024 Aug 31;16(4):208-37. [https://doi.org/10.12731/2658-6649-2024-16-4-1249]

Kind regards
