# Supplementary material for: Multidrug-resistant bacterial profiles of inanimate objects at Zewditu Memorial Hospital, Addis Ababa, Ethiopia
Source: PLoS One. 2025 Oct 7;20(10):e0333361. doi: 10.1371/journal.pone.0333361 (PMC12503313; doi:10.1371/journal.pone.0333361)
Supplement: Attachment — Submitted filename: Comments 2025.docx [file pone.0333361.s003.docx]

This study presents **Multidrug-resistant bacterial profiles of inanimate objects at Zewditu Memorial Hospital, Addis Ababa, Ethiopia.** This finding is relevant for clinical and diagnostic applications.

**Comments**

Line: 72- 73 *Common ESBL- and carbapenemase-producing bacteria in nosocomial infections include Escherichia coli, Klebsiella pneumoniae, P. aeruginosa, and Enterobacter cloacae.* Reference is required

Line: 83-84 Hospital *design and hygiene practices should primarily focus on managing nosocomial pathogens and resistant strains, which can contaminate surfaces, hands, equipment, and air*. This sentence is not required here, please remove it. It is better to use it in the discussion part.

Line: 109: ……………ISO/DIS 14698-1 guidelines. Please cite the guideline

Line 120-123. ---------------------CLSI 34th edition (2024). Please cite the CLSI standard

Line: 133----------------------------CLSI guidelines. Please don’t forget to cite while using the guidelines and Literature

Line 170-171: ………. Please remove this sentence and take it with the figure

Table 3: Abbreviations of Antibiotics should be indicated in full form as a footnote in Table 3

Table 3: Are you sure that AMP susceptibility tests are recommended for K. pneumoniae? I don’t think so. Please be sure and correct the typo.

Table 4: What does **Ptn stand for** in the table? Please write in full form or note in the footnote, please.

Table 4: Please be sure all abbreviations are listed as full forms before being abbreviated, or indicate their full form in the footnote

Lines 24-241: should be removed from here and go with the figure

Line 250-251: Please remember that the topic of figures always goes with the figure. Don’t put it alone in the manuscript. Please take it to the figure.

Line 266-267: Remove the topic and put it in Figure 4

Line 273-274 The topic of the figure is not required here

Line 276: You said that “Several studies ……………..but you only used one reference (14). The term several indicates more than one reference. Could you add more references or modify the sentence, please?

Line 282-283: The rise in antimicrobial resistance (AMR) also contributes to an increased morbidity and mortality rates for HCAIs. This sentence does not make sense. Please rewrite or remove it.
