# Supplementary material for: Multidrug-resistant bacterial profiles of inanimate objects at Zewditu Memorial Hospital, Addis Ababa, Ethiopia
Source: PLoS One. 2025 Oct 7;20(10):e0333361. doi: 10.1371/journal.pone.0333361 (PMC12503313; doi:10.1371/journal.pone.0333361)
Supplement: Attachment — Submitted filename: Response to Reviewers.docx [file pone.0333361.s005.docx]

June 06/2025

Dear Editor; PLOS ONE

Thank you very much for allowing us to revise our manuscript entitled “Multidrug-resistant bacterial profiles of inanimate objects at Zewditu Memorial Hospital, Addis Ababa, Ethiopia.”. We value the reviewers' insightful comments and have thoughtfully considered and responded to each one. Our thorough, point-by-point answers are included in the attachment below. The reviewers' comments have been copied and pasted, and the author's responses are italicized. We have made a number of appropriate changes to the article in this revision, which are indicated in the updated manuscript through track changes. We believe that our paper has been greatly enhanced by the adjustments we made in response to the reviewers' feedback. We sincerely thank the reviewers for their insightful comments and helpful critiques of our article.

With best regards;

Hiwot Lidet;

Corresponding Author

**Point-by-point responses to the reviewers’ comments**

**Reviewer 1**

Reviewer #1: Dear author,

1- References 9, 21, 37, and 40 are old, please use new references.

**Author’s response**: *Thank you very much for your suggestion;* *we have replaced reference number 9 & 40 and removed reference number 21 and 37.*

2- Please insert the suitable footnotes for Figures2, 3 and 5.

**Author’s response**: *Thank you for your comments; we have added the suitable footnotes as you have suggested.*

3- Please insert the suitable footnotes for Tables 3, 4 and 5.

**Author’s response**: *Thank you for your comments; we have added the suitable footnotes for our tables as you have suggested.*

4- Do you have information about false positives or false negatives of samples?

**Author’s response**: *Thank you for your question; however, there is no information about false positives and false negatives, since the hospital environment samples were processed immediately after collection.*

Kind regards

**Reviewer #2: Manuscript Number: PONE-D-25-12506**

Title: Multidrug-resistant bacterial profiles of inanimate objects at Zewditu Memorial Hospital, Addis Ababa, Ethiopia.

Overview and general recommendation:

This subject is very important because in developing countries, there is a lack of information on the extent and types of contamination, as well as the microbiological profiles of commonly used medical instruments and inanimate surfaces in hospitals. Therefore, ongoing research in this area is necessary to address and mitigate the problem. I found the manuscript well written, well described and the references were new. I listed minor points below for editing.

Comments to Authors:

Minor points

1- In this manuscript: you wrote (12 times Our)!!! The rule of manuscript writing is to avoid using (Our). So you should delete (Our), use the formal scientific words (This review or The current review or The present review).

**Author’s response**: *Thank you very much for your suggestion;* *we have omitted the word “our’’ and substitute it with relevant scientific words.*

2- In 364 line; you wrote (We)!!! The rule of manuscript writing is to avoid using (We). So you should delete (We), use the formal scientific words (This review or The current review or The present review).

**Author’s response**: *Thank you very much for your comment and suggestion;* *we have omitted the word “We’’ and substitute it with relevant scientific word in line number 366.*

2- In discussion: 366line; I very recommend you to add new references here related with [Hospital-acquired infections and carbapenemase-producing isolates with gene transfer or mutations] to be stronger scientifically. Kindly add the two mentioned new references Hasan SA, Raoof WM, Ahmed KK. Antibacterial activity of deer musk and Ziziphus spina-christi against carbapebem resis-tant gram negative bacteria isolated from patients with burns and wounds. Regulatory Mechanisms in Biosystems. 2024 Apr 17;15(2):267-78. [DOI: <https://doi.org/10.15421/022439>]

Hasan SA, Raoof WM, Ahmed KK. First Report Of Co-Harboring Bleomycin Resistance Gene (Blembl) And Carbapenemase Resistance Gene (Blandm-1) Klebsiella Pneumoniae In Iraq With Comparison Study Among The Sensitivity Test, The Bd Phoenix Cpo Detect Test, And The Rapidec® Carba Np Test. Siberian Journal of Life Sciences and Agriculture. 2024 Aug 31;16(4):208-37. [https://doi.org/10.12731/2658-6649-2024-16-4-1249]

**Author’s response**: *we would like to thank you for your recommendation;* *we have included those references in line number 388.*

Kind regards

**Reviewer #3**: This study presents Multidrug-resistant bacterial profiles of inanimate objects at Zewditu Memorial Hospital, Addis Ababa, Ethiopia. This finding is relevant for clinical and diagnostic applications.

**Comments**

Line: 72- 73 Common ESBL- and carbapenemase-producing bacteria in nosocomial infections include *Escherichia coli, Klebsiella pneumoniae, P. aeruginosa*, and *Enterobacter cloacae*. Reference is required

**Author’s response**: *Thank you very much for your suggestion;* *we have added a reference to the line number 72-73.*

Line: 83-84 Hospital design and hygiene practices should primarily focus on managing nosocomial pathogens and resistant strains, which can contaminate surfaces, hands, equipment, and air. This sentence is not required here, please remove it. It is better to use it in the discussion part.

**Author’s response**: *Thank you very much for your comment;* *we have moved the sentence into the discussion part, line number 385-388.*

Line: 109: ……………ISO/DIS 14698-1 guidelines. Please cite the guideline

**Author’s response**: *Thank you very much for your comment;* *we have cited the sentence with reference.*

Line 120-123. ---------------------CLSI 34th edition (2024). Please cite the CLSI standard

**Author’s response**: *Thank you very much for your suggestion;* *we have cited the sentence with reference.*

Line: 133----------------------------CLSI guidelines. Please don’t forget to cite while using the guidelines and Literature

**Author’s response**: *Thank you very much for your suggestion;* *we have cited the sentence with reference.*

Line 170-171: ………. Please remove this sentence and take it with the figure

**Author’s response**: *Thank you very much for your suggestion;* *we have moved the sentence into the figure.*

Table 3: Abbreviations of Antibiotics should be indicated in full form as a footnote in Table 3

**Author’s response**: *Thank you very much for your suggestion;* *we have added the full forms of antibiotics as a footnote in Table 3.*

Table 3: Are you sure that AMP susceptibility tests are recommended for *K. pneumoniae*? I don’t think so. Please be sure and correct the typo.

**Author’s response**: *Thank you very much for your question, but yes CLSI guideline recommends testing AMP for Enterobacteriaceae that includes K. pneumoniae.*

Table 4: What does Ptn stand for in the table? Please write in full form or note in the footnote, please.

**Author’s response**: *Thank you very much for your question and suggestion. We have written it in full form in the table.*

Table 4: Please be sure all abbreviations are listed as full forms before being abbreviated, or indicate their full form in the footnote

**Author’s response**: *Thank you very much for your suggestion. We have written all abbreviation in full form.*

Lines 24-241: should be removed from here and go with the figure

**Author’s response**: *Thank you very much for your suggestion;* *we have moved the sentence into the figure.*

Line 250-251: Please remember that the topic of figures always goes with the figure. Don’t put it alone in the manuscript. Please take it to the figure.

**Author’s response**: *Thank you very much for your suggestion;* *we have moved the sentence into the figure.*

Line 266-267: Remove the topic and put it in Figure 4

**Author’s response**: *Thank you very much for your suggestion;* *we have moved the sentence into the figure.*

Line 273-274 The topic of the figure is not required here

**Author’s response**: *Thank you very much for your suggestion;* *we have moved the sentence into the figure.*

Line 276: You said that “Several studies ……………..but you only used one reference (14). The term several indicates more than one reference. Could you add more references or modify the sentence, please?

**Author’s response**: *Thank you very much for your comment;* *we have modified the word in the sentence.*

Line 282-283: The rise in antimicrobial resistance (AMR) also contributes to an increased morbidity and mortality rates for HCAIs. This sentence does not make sense. Please rewrite or remove it.

**Author’s response**: *Thank you very much for your suggestion;* *we have removed the sentence.*
