# Supplementary material for: Multidrug-resistant bacterial profiles of inanimate objects at Zewditu Memorial Hospital, Addis Ababa, Ethiopia
Source: PLoS One. 2025 Oct 7;20(10):e0333361. doi: 10.1371/journal.pone.0333361 (PMC12503313; doi:10.1371/journal.pone.0333361)
Supplement: Attachment — Submitted filename: Response_to_Reviewers_auresp_2.docx [file pone.0333361.s006.docx]

August 25/2025

Dear Editor; PLOS ONE

Thank you very much for allowing us to revise our manuscript entitled “Multidrug-resistant bacterial profiles of inanimate objects at Zewditu Memorial Hospital, Addis Ababa, Ethiopia.”. We value the reviewers' insightful comments and have thoughtfully considered and responded. Our thorough answer is included in the attachment below. The reviewers' comments have been copied and pasted, and the author's responses are italicized. We have made changes to the references of the article in this revision, which are indicated in the updated manuscript through track changes. We sincerely thank the reviewers for their insightful comments of our article.

With best regards;

Hiwot Lidet;

Corresponding Author

**Responses to the reviewers’ comments**

**Reviewer 1**

Reviewer #1: Dear author,

This is a good article and could provide useful information for readers of this article. Please make the following corrections. References 24 and 44 are old, please use new references.

**Author’s response**: *Thank you very much for your comment;* *we have removed those references since they are old.*

Kind regards.

**Reviewer 2**

Reviewer #2: Greetings

Very good work.

You did all the scientific requirements by the correct way.

Now, your manuscript is more accurate.

Kind regards.

**Author’s response**: *Thank you very much.*
